# SOCS1 Mimetics and Antagonists: A Complementary Approach to Positive and Negative Regulation of Immune Function

**DOI:** 10.3389/fimmu.2015.00183

**Published:** 2015-04-21

**Authors:** Chulbul M. I. Ahmed, Joseph Larkin, Howard M. Johnson

**Affiliations:** ^1^Department of Microbiology and Cell Science, University of Florida, Gainesville, FL, USA

**Keywords:** immune therapy, SOCS mimetics, SOCS antagonist, antivirals, poxvirus

## Abstract

Suppressors of cytokine signaling (SOCS) are inducible intracellular proteins that play essential regulatory roles in both immune and non-immune function. Of the eight known members, SOCS1 and SOCS3 in conjunction with regulatory T cells play key roles in regulation of the immune system. Molecular tools such as gene transfections and siRNA have played a major role in our functional understanding of the SOCS proteins where a key functional domain of 12-amino acid residues called the kinase inhibitory region (KIR) has been identified on SOCS1 and SOCS3. KIR plays a key role in inhibition of the JAK2 tyrosine kinase, which in turn plays a key role in cytokine signaling. A peptide corresponding to KIR (SOCS1-KIR) bound to the activation loop of JAK2 and inhibited tyrosine phosphorylation of STAT1α transcription factor by JAK2. Cell internalized SOCS1-KIR is a potent therapeutic in the experimental allergic encephalomyelitis (EAE) mouse model of multiple sclerosis and showed promise in a psoriasis model and a model of diabetes-associated cardiovascular disease. By contrast, a peptide, pJAK2(1001–1013), that corresponds to the activation loop of JAK2 is a SOCS1 antagonist. The antagonist enhanced innate and adaptive immune response against a broad range of viruses including herpes simplex virus, vaccinia virus, and an EMC picornavirus. SOCS mimetics and antagonists are thus potential therapeutics for negative and positive regulation of the immune system.

## Introduction

The immune system consists of both positive and negative regulators that act in harmony to maintain immune homeostasis. A family of intracellular proteins called suppressors of cytokine signaling (SOCS) negatively regulates both receptor-associated tyrosine kinases such as the JAKs and receptor tyrosine kinases such as EGF receptor (1–5). The JAKs play a key role in cytokine signaling by phosphorylation and activation of transcription factors called STATs (6). As these tyrosine kinases thus play key roles in cytokine, growth factor, and hormone signaling, various SOCS play an indispensable role in regulation of their activities (7, 8). The SOCS group has a membership of eight, SOCS1, SOCS2, SOCS3, SOCS4, SOCS5, SOCS6, SOCS7, and SH2 cytokine-inducible protein. SOCS1 is important for its regulation of the JAK2 tyrosine kinase as well as the related kinases TYK2 and JAK1 (2, 8). Receptor tyrosine kinases such as EGF receptor are also regulated by SOCS1 (4). Deficiency in SOCS1 as per knockout of the gene (SOCS1^−/−^) results in a neonatal fatal inflammatory disease where the cytokine gamma interferon (IFNγ) is the chief cause of the resultant pathology (9).

Among other regulatory players in the immune response, regulatory T cells (Tregs), particularly so-called natural Tregs, also play a key role in immune homeostasis (10–13). We and others have shown that there is an obligatory cross-talk between SOCS1 and peripheral Tregs where SOCS1 plays a key role in maintaining Treg stability (12–14). Molecular genetics via transfections and siRNA approaches have generally been the only way to regulate the intracellular activities of SOCS such as SOCS1 (15). SOCS1 protein has not lent itself to a functional approach to immune regulation for various technical reasons; however, SOCS3 protein has been obtained and used in structure studies of SOCS3/JAK2 interaction (16). We have, by contrast, been able to develop small peptide mimetics and antagonists of SOCS1 (17–19). This has been achieved for the following reasons. First, we have identified one of the key regions of the SOCS1 molecule called the kinase inhibitory region (KIR) as a target for development of SOCS1 mimetics (17). Having done that, we focused on the JAK2 tyrosine kinase activation or autocatalytic region, which interacts with the SOCS1 mimetic as a target for development of SOCS1 (and SOCS3) antagonists (18). Second, it has been well established among the structural biology community that many key proteins, particularly those associated with signaling, lack stable tertiary structure yet carry out numerous biological functions (20). The KIRs of SOCS1 and SOCS3 are slightly homologous and solution structure of SOCS3 has shown that KIR is unstructured (21). The activation loop of JAK2 is similarly unstructured (20). We reasoned that unstructured sequences of proteins are candidates for intrinsic function independent of other regions of the proteins. With this understanding, we were not conceptually restricted in the development of small peptide mimetics and antagonists of SOCS1. We show here that the SOCS1 mimetics are effective in treatment of autoimmune and inflammatory disorders and that the SOCS1 antagonists enhance the immune response for effective handling of viral infections by tilting immune homeostasis toward increased immune activity. This review/perspective thus demonstrates the success of our approach.

## Development of SOCS1 Mimetics

We focused on the activation loop of JAK2 to first develop inhibitors of its enzymatic activity independent of the physiological regulation of JAKs by SOCS. The autophosphorylation or activation loop region of human JAK2 became a focal point in our development of SOCS1 mimetics. The amino acid sequence of this region is ^1001^LPQDKEYYKVKEP for human JAK2 and is depicted symbolically as pJAK2(1001–1013) with the p of pJAK2 indicative of phosphorylation of tyrosine 1007, reflecting its activation state (22). Our first SOCS1 mimetic was developed using an algorithm based on hydropathic complementarity to the JAK2 activation loop (23, 24). We thus synthesized a 12-amino acid peptide, WLVFFVIFYFFR, and determined that it specifically bound the activation loop of JAK2, inhibited IFNγ activation of JAK2 and STAT1α as well as phosphorylation of the IFNγ receptor subunit IFNGR1 (4). Functionally, the tyrosine kinase inhibitory peptide (Tkip) inhibited antiviral activity of IFNγ as well as upregulation of MHC class I molecules on fibroblast cells (4). When compared to the KIR residues of SOCS1, Tkip showed homology with the KIR residues F56, F59, and R60 (22). F59 has been shown to be critical for binding of SOCS1 to activated JAK2 (22). The sequences of Tkip and SOCS1-KIR are shown aligned in Table 1 to illustrate the critical residues.

**Table 1:** **SOCS1 mimetics and antagonists**.

Peptide	Function	Sequence	Reference
Tkip	SOCS1 mimetic	WLVFFVI**F**Y**FFR**	(4)
SOCS1-KIR	SOCS1 mimetic	^53^DTH**F**RT**FR**SHSDYRRI	(18)
SOCS3-KIR	Pseudosubstrate	^20^LRLKTFSSKSEYQLVV	(16)
pJAK2(1001–1013)	SOCS1 antagonist	^1001^LPQDKEpYYKVKEP	(18)

*Tyrosine kinase inhibitory peptide (Tkip) was developed based on hydropathic complementarity to the activation loop of the tyrosine kinase JAK2 (16). pJAK2(1001–1013) is a peptide that corresponds to the activation loop of JAK2. pJAK2 indicates the activation state with phosphorylated Y1007. The kinase inhibitory region (KIR) of human SOCS1 is represented by peptide SOCS1-KIR. The KIR region of SOCS3 is represented by the peptide SOCS3-KIR. Tkip and SOCS1-KIR are aligned to illustrate homologous residues in bold. F56 and F59 have been shown to be critical for SOCS1 binding to JAK2 (22)*.

The KIR region of SOCS1 and SOCS3 are located in the N-terminus of the proteins and are adjacent to a similarly short extended SH2 sequence (ESS). C-terminus to ESS is the SRC homology 2 (SH2) domain, followed by the 40 amino acid SOCS box (3). The SOCS box is shared in all SOCS proteins, in contrast to the restriction of KIR to SOCS1 and SOCS3. The SOCS box is involved in proteasomal degradation of the associated tyrosine kinases. Thus, SOCS1-KIR only inhibits JAK2 kinase activity, while the intact SOCS1 protein inhibits JAK2 kinase activity and initiates its proteasomal degradation.

Peptides corresponding to the 12-amino acid KIR (SOCS1-KIR) and the adjacent extended SH2 sequence (ESS) regions of SOCS1 were compared to Tkip for binding to JAK2 activation loop, pJAK2(1001–1013). Both Tkip and SOCS1-KIR, but not the ESS peptide, bound to pJAK2(1001–1013). Tkip, thus, fortuitously led us to the KIR region of SOCS1 as a potential SOCS1 mimetic. Dose–response competitive binding suggested that Tkip and SOCS1-KIR similarly recognized the activation loop as per the pJAK2(1001–1013) peptide. Tkip inhibited JAK2 autophosphorylation; SOCS1-KIR did not. Both inhibited STAT1α activation, IFNγ activation of macrophages, and antigen-specific lymphocyte proliferation. The use of the SOCS1 mimetics in treatment of autoimmune diseases in mouse models is presented under a separate section below.

Remarkable progress has been made in structure studies of SOCS3, which also contains a KIR region in its N-terminus (3). Care should be exercised, however, in the extrapolation of the structure findings with SOCS3 to SOCS1, particularly concerning KIR. In crystallographic determination of the structure of SOCS3/JAK2 complex, it was shown that KIR was required for complex formation, but the SOCS3-KIR peptide alone did not block JAK2 kinase activity (16). In the same experiment, however, the authors did show that SOCS1-KIR blocked JAK2 activity, which confirms our original observation that SOCS1-KIR peptide mimics SOCS1 protein in inhibiting JAK2 (18). For SOCS3, KIR is thus described as a “pseudosubstrate” in that it is thought to block substrate access to the activation loop of JAK2 (16). Since SOCS3-KIR is required for SOCS3 binding to JAK2, it seems reasonable to suppose that it is binding to something on JAK2. Since the crystallographic studies showed that unstructured KIR and JAK2 activation loop were in close proximity, it is possible that SOCS3-KIR could bind to the activation loop in such a manner that would not block JAK2 kinase activity, but would block substrate access, possibly in concert with another region(s) of SOCS3.

## Development of SOCS1 Antagonist

**Figure 1:**
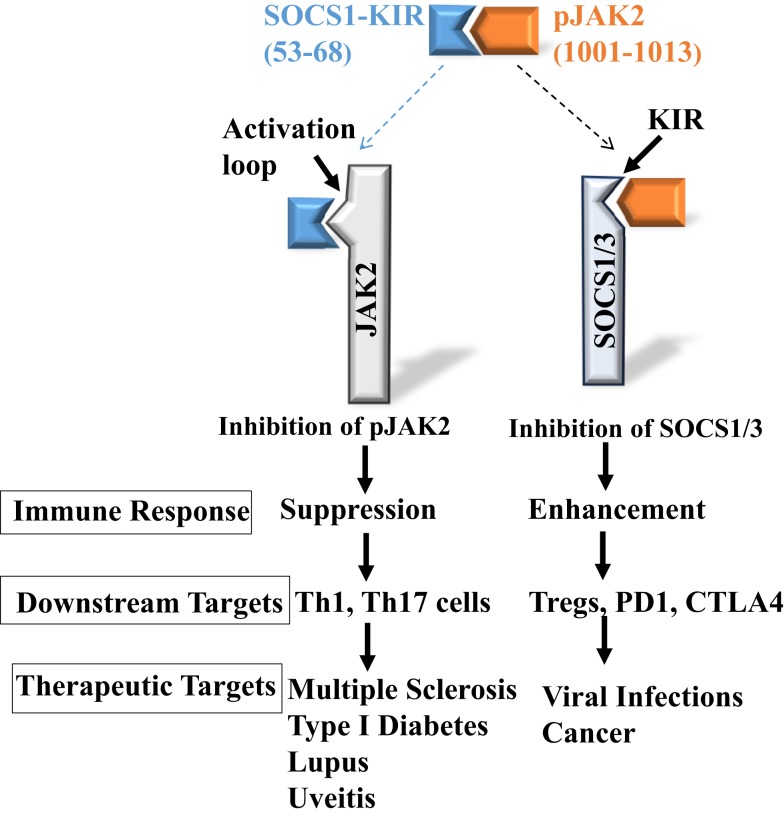
**Development of SOCS1 mimetic and antagonist**. Peptides corresponding to SOCS1-KIR, SOCS1-KIR (53–68), and pJAK2 activation loop, pJAK2(1001–1013), interact with each other, demonstrating complementarity. They also interact with the cognate SOCS1 and pJAK2 proteins, respectively, and inhibit pJAK2 and SOCS1 functions, resulting in either suppressed or enhanced immune response.

Both SOCS1 and SOCS3-KIR are required for the binding of those SOCS to JAK2 as well as TYK2 and JAK1 tyrosine kinases (3). As indicated, SOCS1-KIR can inhibit JAK2 kinase activity, and although SOCS3-KIR functions as a pseudosubstrate, it does not inhibit JAK2 in the absence of other domains of SOCS3. In any event, both the KIRs of SOCS1 and SOCS3 are intimately associated with the activation loop of JAK2 as reflected in the JAK2 peptide of Table 1. In the activated state, the JAK2 kinase domain is associated with the pseudokinase domain, suggesting that this association is responsible for JAK2 autoinhibition. Importantly, the activation loop is in the kinase domain and phosphorylation of Y1007 in the activation loop is associated with activation of JAK2 (16). Thus, identification of the SOCS1-KIR binding site on JAK2 leads conceptually to development of a SOCS1 antagonist for enhancement of the immune response. As indicated above, JAK2 activation loop peptide, pJAK2(1001–1013), binds SOCS1-KIR. Cell-penetrating lipo-pJAK2(1001–1013) increased IFNγ and IL-6 biological activity by blocking SOCS1 function in cells (18). Consistent with increasing IFNγ activity, JAK2 activation loop peptide enhanced IFN activity against herpes simplex virus-1 (HSV-1) in keratinocytes (25). The anti-HSV-1 effects of the SOCS1 antagonist is expanded upon in the antiviral section below with respect to other viruses in both cultures and animal models. The development of SOCS1 mimetic and antagonist is illustrated in Figure 1.

## Cross-Talk between SOCS1 and Regulatory T Cells

Forkhead box P3 (FoxP3) natural or constitutive regulatory T cells (Tregs) play an essential role in homeostatic regulation as FoxP3 Treg-deficient mice (scurfy) die of inflammatory disease by 3 weeks after birth (13). The intracellular SOCS proteins, particularly SOCS1, similarly play an indispensible role in such disorders. In this regard, SOCS1 knockout (SOCS1^−/−^) mice uniformly suffer neonatal death by 3 weeks after birth, similar to the case for FoxP3 Treg deficiency, with unregulated IFNγ playing the central role in the malignant inflammation that is responsible for the fatal outcome (9). Thus, despite their absolute requirement for normal development, there has been little interest concerning the possibility of cross-talk between Tregs and SOCS1. Intuitively, it would seem that such communication is necessary; otherwise, one should expect difficulties in terms of maintaining lymphocyte homeostasis.

We and others have recently shown that SOCS1 and FoxP3 Tregs do indeed interact in such a way that is particularly beneficial to Tregs. Specifically, SOCS1^−/−^ mice were deficient in peripheral Tregs despite enhanced thymic development (10–12). Adoptive transfer of CD4^+^ T lymphocytes that express the SOCS1 gene or parental administration of the SOCS1 mimetic SOCS1-KIR each induced significant but short-term survival of SOCS1^−/−^ mice. However, adoptive transfer of the CD4^+^ T cells combined with administration of SOCS1-KIR to SOCS1^−/−^ neonatal mice resulted in increased survival long term (12). In contrast to the periphery, FoxP3 expression in thymic lymphocytes was similar in wild-type and SOCS1^−/−^ mice. Consistent with increased survival, combined CD4^+^/SOCS1-KIR treatment resulted in decreased leukocyte organ infiltration, reduction in serum IFNγ, and enhanced accumulation of FoxP3 Tregs in SOCS1^−/−^ mice. These data suggest that SOCS1 is required for normal peripheral FoxP3 Treg function, and in fact may play a hierarchical role in SOCS1/Treg cross-talk as stable expression of FoxP3 in Tregs is dependent on SOCS1 in these cells (14).

Consistent with the above, it has been shown that SOCS1^−/−^ Tregs produce high levels of IFNγ and rapidly lose FoxP_3_ when transferred into immunodeficient Rag2^−/−^ mice or when cultured *in vitro* (14). The SOCS1^−/−^ Tregs showed hyperactivation of transcription factors STAT1 and STAT3, and it has been proposed that such activation is responsible for Treg instability and loss of suppressive functions (14). How STAT activation is mechanistically linked to loss of FoxP3 and Treg instability is, however, not known.

There is evidence that a subset of Treg cells can convert to a T helper 1 (Th1) or T helper 17 (Th17) phenotype under inflammatory and autoimmune conditions (26). Th1 and/or Th17 cells are the effectors in such diseases as type I diabetes and multiple sclerosis (MS) (17, 27). Thus, Treg cells may initially respond to control an inflammatory or autoimmune state but then undergo conversion and actually exacerbate the condition. Focus on the E2 ubiquitin-conjugating enzyme Ubc13 has provided some insight into Treg plasticity (26). Ubc13 is involved in the formation and conjugation of lysine 63-linked polyubiquitin chains to phosphorylated inhibitor of NF-κB (IκB) where phosphorylation is mediated by IκB kinase (IKK) (26). IκB is then separated from NF-κB, freeing NF-κB to carry out specific transcription. Mice that had Ubc13 specifically ablated or knocked out in Treg cells suffered from systemic autoimmunity with reduction in weight and inflammatory lymphocyte infiltration of the heart, kidney, liver, and lung. Ubc13-deficient Treg cells were shown to be capable of causing the autoimmune condition. Related to this, Ubc13-deficient Treg cells were defective in SOCS1 and IL-10 induction. Reporter gene assays showed that active NF-κB was required for SOCS1 induction but Ubc13 ablated cells lacked active NF-κB because of lack of an effect on IκB. Treatment of cells with the SOCS1 mimetic SOCS1-KIR suppressed IL-17 production in cells from Ubc13-deficient mice. Further, loss of weight and a normal T cell profile were partially restored in SOCS1-KIR treated mice. This study thus showed that Ubc13 plays a critical role in preventing Treg cells from undergoing harmful phenotype changes and that Ubc13 regulated downstream signaling via SOCS1 is key to maintaining Treg cell homeostasis. Translationally, it suggests a role for SOCS1 mimetics in treating inflammatory and autoimmune diseases where Ubc13-like dysregulation may be involved.

SOCS1, regulatory T cells, the programed death-1 (PD-1), and T-lymphocyte-associated protein 4 (CTLA-4) immune mediators are all involved in negative modulation of the immune response. As was shown above with SOCS1 and Tregs, it appears that all of these regulatory players including SOCS1 are interconnected and interdependent, probably in complex ways. It was recently shown, for example, that there is cross-talk between SOCS1 and PD-1, where siRNA silencing of SOCS1 expression resulted in inhibition of PD-1 upregulation (28). Similarly, CTLA-4 has been shown to be a key effector molecule in Treg function (29, 30). The modulatory effect of SOCS1 mimetic and antagonist on Tregs, thus, probably extends to an effect on these other players in positive and negative regulation of immune function (see Figure 1 for example). In principle, this suggests a global approach to positive and negative regulation of immune functions via the SOCS1 mimetic and antagonist. Currently, specific reagents are used to attack various players such as PD-1 and CTLA-4 for enhancement of immune system against cancer (31). Theoretically, the SOCS1 antagonist should affect these molecules along with its effects on Tregs.

## Effect of SOCS1-KIR in Autoimmunity: The Experimental Allergic Encephalomyelitis Model as Well as Other Autoinflammatory Disease Models

In the study of the possible role of SOCS1 and/or SOCS3 in the therapeutic efficacy of IFNβ in the treatment of relapsing–remitting MS, astrocytes treated with IFNβ showed upregulation of SOCS1 and SOCS3 (32). This upregulation was due to the corresponding activation of STAT1α and STAT3 by SOCS1 and SOCS3, respectively. The chain of events affected chemokine production and lymphocyte infiltration of the central nervous system (CNS). Given that IFNβ is an effective therapeutic for relapsing–remitting MS (33, 34), it is possible that SOCS1 and SOCS3 play an important role in the effectiveness. In a model of Th17-mediated experimental allergic encephalomyelitis (EAE), the loss of SOCS1 in T cells resulted in increased IFNγ activity and a shift of the T cell population from Th17 to Th1, thus alleviating the EAE (35). In our mouse model of EAE where both Th1 and Th17 cells were involved, loss of SOCS1 from T cells did not protect from EAE (17). Specifically, quantitative reverse transcription polymerase chain reaction (qRT-PCR) results for SOCS1 and SOCS3 mRNA profiles in CNS infiltrating T cells showed a complex pattern. For example, SOCS1 mRNA levels were absent, while SOCS3 levels were modestly increased. By comparison, splenic CD4^+^ T cell profiles of mRNA were modest for both SOCS1 and SOCS3. Significantly, non-CD4^+^ monocytic cells showed a high level of SOCS3 in the CNS relative to that of SOCS1. Thus, endogenous SOCS may help control cells like macrophage, microglia, or dendritic cells in the CNS, while the infiltrating effector T cells, not dependent on the accessory cells (36), may be little affected due to the absence of endogenous SOCS1 and/or SOCS3. SOCS mimetics could potentially augment or replace the reduced endogenous SOCS activity of CNS infiltrating lymphocytes and thus have a therapeutic effect in EAE.

We have shown that the Tkip SOCS1 mimetic is therapeutic against relapsing–remitting EAE in mice, dampening both the cellular and humoral immune responses against myelin basic protein (MBP) (37). In a more comprehensive study of treating EAE mice with SOCS1-KIR, we similarly showed SOCS1 mimetic therapeutic protection (17). Mice had lymphocyte infiltration of the CNS at the beginning of treatment, but such infiltration was cleared after 3 weeks of treatment. Both CD4+ Th1 and Th17 cells were suppressed, as well as the IFNγ and IL-17A cytokines are, respectively, associated with them. We further showed for the case of Th17 cells that the polarizing cytokine IL-23 was inhibited by SOCS-KIR. There is evidence that Th1 and Th17 cells function cooperatively in the pathology of EAE where Th1 cells compromise the blood–brain barrier (BBB) and facilitate infiltration by Th17 cells (38, 39). The restoration of a pathologic brain to a normal state by SOCS1-KIR in our EAE model is consistent with its inhibitory effects on both the Th1 and Th17 arms of EAE and possibly MS.

SOCS1-KIR or its analogs also show promise in other autoimmune and/or inflammatory disorders. Psoriasis is an autoinflammatory disorder of the skin that involves interaction between cells of the immune system with keratinocytes of the skin [reviewed in Ref. (40)]. Both the innate and adaptive immune systems play a role in the pathogenesis of psoriasis. Under homeostatic conditions, microbial attack of the skin results in activation of innate immune cells such as dendritic cells, which produce the interleukins 12 (IL-12) and 23 (IL-23), which are involved in the activation of Th1 and Th17 cells, respectively. Keratinocytes in their capacity as innate immune cells play a role in dendritic cell activation as per their production of IL-1, IL-6, and tumor necrosis factor α (TNFα). Th1 and Th17 cells in turn act on keratinocytes via IFNγ (Th1) and IL-17A and F (Th17). This further activates the keratinocytes to produce chemokines, interleukins, and other proteins that have both direct and indirect effect on the microbial insult. Psoriasis is thought to develop because of a dysregulated feedback loop between the innate and adaptive immune systems. Psoriasis can be controlled but the dysregulation cannot be converted back to the homeostatic state.

An understanding of the role of SOCS1 and/or SOCS3 in the pathogenesis of psoriasis is in its infancy. It has been reported that keratinocytes with deleted SOCS3, but not SOCS1 in mice resulted in psoriasis-like skin pathology (41). Others have reported that SOCS1 plays a key role in Th1 and IFNγ-induced form of psoriasis-like skin disorder in experiments where human keratinocytes were transiently transfected with SOCS1 genes (42).

Treatment of human keratinocytes or human skin explants with IFNγ in an experimental setting produced an inflammatory pattern similar to that of psoriasis with JAK2 involvement in activation of STAT1α (43). This in turn induced the epidermal expression of the integrin ICAM-1, HLA-DR MHC, and chemokines CXCL10 and CCL2. Immunohistochemically, the IFNγ-treated explants were similar to those from psoriasis patients. The SOCS1-KIR mimetic analog significantly blocked STAT1α activation as well as expression of the integrin, MHC, and chemokines. It was proposed that the SOCS mimetic was a potential therapeutic for psoriasis (43).

Obesity is a major problem in the United States as well as the world at large (44). It is associated with a condition called metabolic syndrome, which is characterized by insulin resistance reviewed in Ref. (45). The adipose tissue associated with the metabolic syndrome fuels the activation of macrophages, which in turn play a major role in the systemic inflammation that affects key tissues and organs such as the liver. The inflammatory cytokines that result from the systemic inflammation cause insulin resistance by inhibiting insulin receptor substrates (IRSs) activity, which are the mediators of insulin signaling via interaction with the insulin receptor tyrosine kinase.

SOCS1 and SOCS3 have been shown to play a paradoxical role in insulin activity in type 2 diabetes. Evidence suggests that these SOCS dampen the inflammatory response associated with metabolic syndrome and thus play a role in increasing insulin sensitivity (45). The flip side of this is that SOCS1 (as well as SOCS3) is also associated with insulin resistance via its competition with IRS 1 and 2 for binding sites on the insulin receptor, through its targeting of IRS1/2 and insulin receptor for proteasomal degradation, and through its inhibitory effect on insulin receptor and JAK kinase activities (46–49).

A recent quite interesting use of SOCS1-KIR was in the treatment of streptozotocin-induced diabetes in a mouse model (27). SOCS1 mimetic was particularly effective in reducing vascular plaque accumulation of lipid, macrophages, and T cells. The atheroprotective effect was accompanied by systemic reduction in proinflammatory Ly6C^high^ monocytes as well as local reduction in aorta expression of chemokines and cytokines. Thus, SOCS1-KIR and other related SOCS mimetics have therapeutic potential to retard the vascular problems associated with diabetes (27). Unlike SOCS1, SOCS1-KIR lacks the ability to target IRS for proteasomal degradation, since it lacks the required SOCS box (3). This could possibly play a role in its effectiveness in reducing the vascular inflammation in the mouse model of diabetes (27).

## Broad Antiviral Activity of SOCS1 Antagonist

There is a constitutive presence of IFNβ (50, 51) and SOCS1 (19) in cells. The constitutive IFNβ plays a key role in optimizing the activity of induced types I and II IFNs (19, 50, 51). At the same time, the constitutive IFNβ as well as induced and added IFNs are regulated by SOCS1 (19). For example, if we treat cells with lipo-pJAK2(1001–1013), we decrease constitutive and induced SOCS1, which in turn increases the levels and activities of the constitutive, induced, and added IFNs. Our demonstration that keratinocytes treated with lipo-pJAK2(1001–1013) possessed enhanced antiviral activity against HSV-1 (25) supported our suppositions about antiviral activity, particularly *in vivo*, against disparate viruses such as vaccinia virus (19) and encephalomyocarditis virus (EMCV) (19) described below in a stringent test of broad, effective antiviral activity.

### Vaccinia virus

Poxviruses are large, complex, double-stranded DNA viruses that have wrecked havoc on human existence over the ages, because of their innate ability to neutralize the IFN system (52, 53). The smallpox virus is historically responsible for some of the most devastating pandemics in the history of humankind and has been estimated to cause approximately 500 million deaths globally in the past century alone (53). Human monkeypox virus is a zoonotic poxvirus with a clinical presentation similar to that of smallpox. The majority of human monkeypox infections occur in Central Africa and if a more infectious, virulent variant should arise, it could represent a health concern and hysteria similar to that involving the recent Ebola virus epidemic in Africa (54).

**Figure 2:**
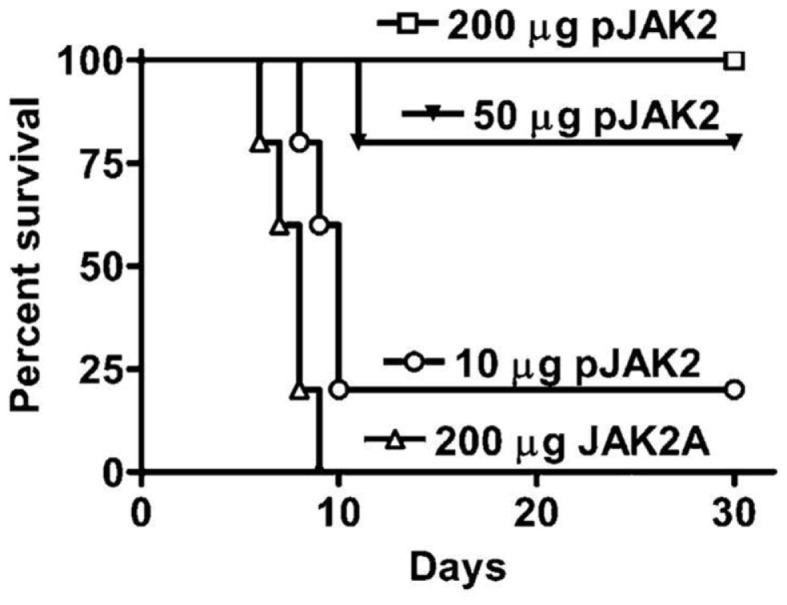
**SOCS1 antagonist is an effective therapeutic for lethal intranasal vaccinia virus infection of C57BL/6 mice**. Lipo-pJAK2(1001–1013), pJAK2 at 10 μg (○), 50 μg (▼), or 200 μg (□) was injected intraperitoneally on days −2, −1, and 0, relative to virus challenge. Lipo-JAK2(1001–1013)2A (JAK2A) has alanine substituted for tyrosines at 1007 and 1008, and is an inactive control. For details, see Ref. (19).

The testing of the SOCS1 antagonist in a vaccinia virus poxvirus model could thus serve two purposes. First, it tests the antiviral effect of the antagonist. Second, it provides a potential much needed drug therapeutic to poxvirus infections. We reasoned that independent of all other therapeutics that the SOCS1 antagonist, lipo-pJAK2(1001–1013), would tilt the immune balance in mice infected with a lethal dose of vaccinia virus toward a more vigorous IFN and innate protective immune response against the virus (19). Antagonist protected mice as shown in Figure 2 in a dose-dependent fashion with complete protection at 200 μg lipo-pJAK2(1001–1013), while controls succumbed to virus by day 9.

The question arises as to whether the SOCS antagonist inhibits virus replication or virus spread. Accordingly, vaccinia virus susceptible cells were treated with lipo-pJAK2(1001–1013) at a final concentration of 50 μM and then challenged with a dose of virus that ensured that all of the cells were infected at the same time, resulting in a one-step growth curve. Lipo-pJAK2(1001–1013) inhibited virus replication by approximately 92% as determined by intracellular virus yield when compared to the control variant lipo-pJAK2(1001–1013)2A. Inhibition was approximately 83% as determined by extracellular virus yield. Thus, SOCS1 antagonist inhibited vaccinia virus replication and not simply its release from the cells. This observation is in stark contrast to inhibition of vaccinia virus release but not replication from cells by two different tyrosine kinase inhibitors (55, 56). Thus, the SOCS1 antagonist inhibited vaccinia virus replication and not simply its release, the latter being less reliable in protecting infected mice.

### Encephalomyocarditis virus

Encephalomyocarditis virus is a plus strand rodent picornavirus but is capable of infecting other species including humans (57). Treatment of L929 fibroblasts with the SOCS1 antagonist at 24 μM prior to infection with EMCV (200 PFU) inhibited EMCV growth by approximately 50% (19). The antagonist variant, by contrast, was only 7% protective. *In vivo* studies involved treatment of C57BL/6 mice with 50, 100, or 200 μg lipo-JAK2(1001–1013) every other day beginning at day 2 and resulted in 60–80% protection at the higher doses. Variant lipo-pJAK2(1001–1013)2A by contrast, was not protective at 200 μg with the death of all the mice by day 5. The extent of protection by the antagonist is remarkable, given the aggressive virulence of EMCV in these experiments. Thus, the SOCS1 antagonist is protective against EMCV as well as against vaccinia virus.

## Conclusion

As key as SOCS proteins such as SOCS1 are to normal immune and non-immune functions, they do not lend themselves to controlled and practical manipulation to restoration of homeostasis in autoimmune and inflammatory disorders. Intrinsically disordered proteins (IDPs) and IDP regions lack stable tertiary structure but are responsible for numerous biological functions, particularly those associated with signaling, transcription, DNA interactions, and cellular division and differentiation (58, 59). We took advantage of this important aspect of protein function in our development of small peptide SOCS1 mimetics based on the KIR region of SOCS1. We similarly developed a SOCS1 antagonist based on the activation loop of JAK2, which is the target of the KIR of SOCS1. We view these peptide mimetics and antagonists as templates of a larger and more general approach to development of mimetics and antagonists of other molecules involved in signaling.

## Author Contributions

CA, performed research; HJ and JL designed research; HJ wrote the manuscript.

## Conflict of Interest Statement

The authors declare that the research was conducted in the absence of any commercial or financial relationships that could be construed as a potential conflict of interest.
